# Multiple Inflammatory Pseudotumors of the Liver Demonstrating Spontaneous Regression: A Case Report

**DOI:** 10.3390/life12010124

**Published:** 2022-01-16

**Authors:** Noriko Ishii-Kitano, Hirayuki Enomoto, Takashi Nishimura, Nobuhiro Aizawa, Yoko Shibata, Akiko Higashiura, Tomoyuki Takashima, Naoto Ikeda, Yukihisa Yuri, Aoi Fujiwara, Kohei Yoshihara, Ryota Yoshioka, Shoki Kawata, Shogo Ota, Ryota Nakano, Hideyuki Shiomi, Seiichi Hirota, Tsutomu Kumabe, Osamu Nakashima, Hiroko Iijima

**Affiliations:** 1 Division of Hepatobiliary and Pancreatic Disease, Department of Internal Medicine, Hyogo College of Medicine, Mukogawa-cho 1-1, Nishinomiya 663-8501, Hyogo, Japan; ishinori1985@yahoo.co.jp (N.I.-K.); tk-nishimura@hyo-med.ac.jp (T.N.); aizawa-n@hyo-med.ac.jp (N.A.); tomo0204@hyo-med.ac.jp (T.T.); nikeneko@hyo-med.ac.jp (N.I.); yu-yukihisa@hyo-med.ac.jp (Y.Y.); ao-fuziwara@hyo-med.ac.jp (A.F.); ko-yoshihara@hyo-med.ac.jp (K.Y.); ri-yoshioka@hyo-med.ac.jp (R.Y.); sh-kawata@hyo-med.ac.jp (S.K.); sy-oota@hyo-med.ac.jp (S.O.); ri-nakano@hyo-med.ac.jp (R.N.); hi-shiomi@hyo-med.ac.jp (H.S.); hiroko-i@hyo-med.ac.jp (H.I.); 2 Ultrasound Imaging Center, Hyogo College of Medicine, Nishinomiya 663-8501, Hyogo, Japan; yoko-p@hyo-med.ac.jp (Y.S.); a-higa@hyo-med.ac.jp (A.H.); 3 Department of Surgical Pathology, Hyogo College of Medicine, Nishinomiya 663-8501, Hyogo, Japan; hiros@hyo-med.ac.jp; 4 Kumabe Clinic, Kikuchi 861-1331, Kumamoto, Japan; ku9li29@yahoo.co.jp; 5 Department of Clinical Laboratory Medicine, Kurume University Hospital, Kurume 830-0011, Fukuoka, Japan; osamu31@med.kurume-u.ac.jp

**Keywords:** inflammatory pseudotumor, spontaneous regression, fluorodeoxyglucose positron emission tomography, contrast-enhanced magnetic resonance imaging, contrast-enhanced computed tomography, contrast-enhanced ultrasonography

## Abstract

Inflammatory pseudotumor (IPT) of the liver is a rare benign disease. IPTs generally develop as solitary nodules, and cases with multiple lesions are uncommon. We herein report a case of multiple IPTs of the liver that spontaneously regressed. A 70-year-old woman with a 10-year history of primary biliary cholangitis and rheumatoid arthritis visited our hospital to receive a periodic medical examination. Abdominal ultrasonography revealed multiple hypoechoic lesions, with a maximum size of 33 mm, in the liver. Contrast-enhanced computed tomography revealed low-attenuation areas in the liver with mild peripheral enhancement at the arterial and portal phases. We first suspected metastatic liver tumors, but fluorodeoxyglucose positron emission tomography, magnetic resonance imaging and contrast-enhanced ultrasonography suggested the tumors to be inconsistent with malignant nodules. A percutaneous biopsy showed shedding of liver cells and abundant fibrosis with infiltration of inflammatory cells. Given these findings, we diagnosed the multiple tumors as IPTs. After careful observation for two months, the tumors almost vanished spontaneously. Physicians should avoid a hasty diagnosis of multiple tumors based solely on a few clinical findings, and a careful assessment with various imaging modalities should be conducted.

## 1. Introduction

Inflammatory pseudotumor (IPT) is a rare benign tumor whose histological findings are characterized by the proliferation of myofibroblastic cells with the infiltration of inflammatory cells, including plasma cells, lymphocytes and macrophages [1,2,3]. Most IPT cases are detected as solitary tumors, and due to a lack of specific clinical and imaging features [1,2,3], IPT is sometimes difficult to discriminate from a malignant tumor. IPTs are most frequently reported as lung tumors, with liver IPTs being uncommon. In addition, spontaneous remission of IPT is rarely reported [4,5,6,7,8,9,10]. 

We herein report a patient with multiple hepatic IPTs that regressed spontaneously in two months. 

## 2. Case Report

A 70-year-old woman with a 10-year history of primary biliary cholangitis (PBC) and rheumatoid arthritis (RA) visited our hospital to receive a regular medical examination, including blood tests and abdominal ultrasonography (US). She did not complain of any subjective symptoms, and no remarkable findings were observed at the physical examinations. 

**Figure 1:**
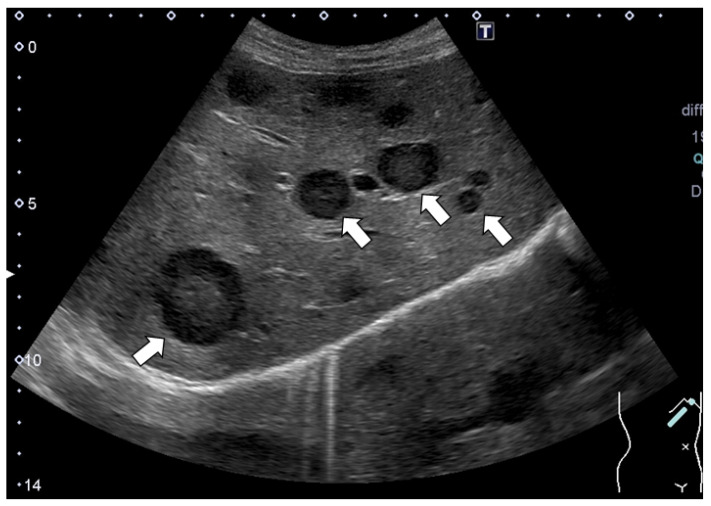
Abdominal ultrasonography (US). Multiple masses with the hypoechoic halo sign at their margins were detected. Some typical tumors with the hypoechoic halo sign are shown with arrows.

Around one month before her visit, she had developed a low-grade fever for a few days; however, no other remarkable episodes were experienced, and she recovered without receiving any new drugs. However, US revealed multiple liver masses with a maximum size of 33 mm, and the tumors had thick hypoechoic areas (halo sign) at their margins (Figure 1). 

**Figure 2:**
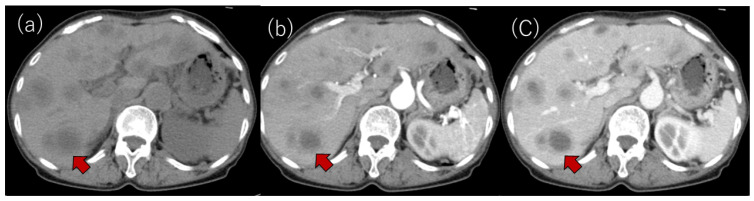
(**a**) Plain CT. Multiple low-attenuation areas were detected; (**b**,**c**) contrast-enhanced CT revealed multiple low-attenuation areas with mild peripheral enhancements at the arterial (**b**) and portal phases (**c**). A typical tumor is shown with an arrow in each panel.

**Figure 3:**
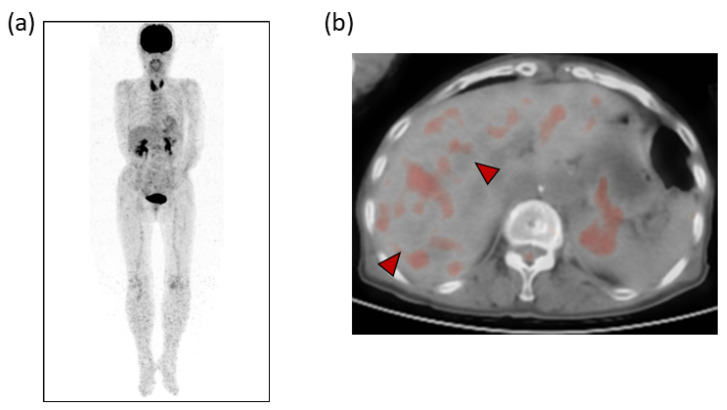
(**a**) ^18^F-fluorodeoxyglucose positron emission tomography (FDG-PET) and (**b**) FDG-PET with CT. No apparently significant signal was observed in the tumors (arrowheads).

Laboratory examinations (Table 1) showed mild elevation of the serum aspartate aminotransferase and C-reactive protein (CRP) levels. The peripheral blood data, including the white blood cell count and platelet count, and the prothrombin time were found to be within the normal range. Tumor markers, such as carcinoembryonic antigen (CEA), carbohydrate antigen 19-9 (CA19-9), α-fetoprotein (AFP) and protein induced by vitamin K absence or antagonist-II (PIVKA-II), were within the normal ranges. Contrast-enhanced computed tomography (CE-CT) showed low-attenuation areas with mild peripheral enhancement at the arterial and portal phase (Figure 2), suggesting the development of metastatic liver tumors. However, no primary tumor candidate was found on the CE-CT. Esophagogastroduodenoscopy and colonoscopy did not reveal any malignant disease. In addition, despite the presence of suspected metastatic tumors on the US and CT, ^18^F-fluorodeoxyglucose positron emission tomography with CT (FDG-PET/CT) showed no significant uptake in the tumors (Figure 3).

**Figure 4:**
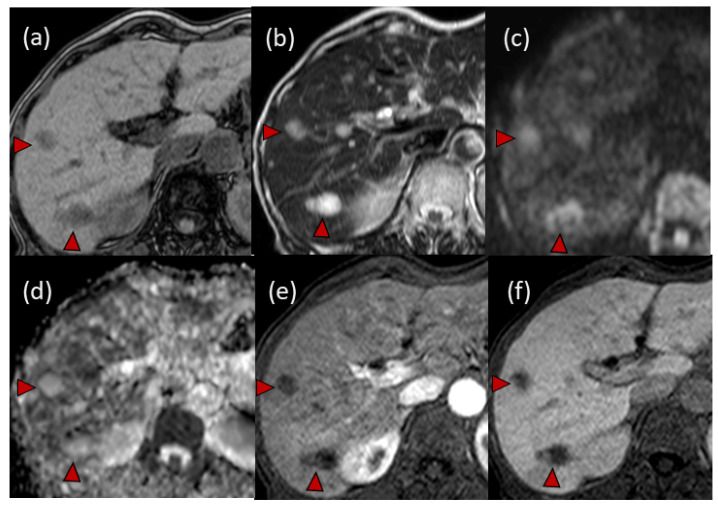
Findings of magnetic resonance imaging (MRI): (**a**) hypo-intensity lesions on T1-weighted imaging; (**b**) high-intensity masses on T2-weighted imaging; (**c**) high-intensity lesions on diffusion-weighted imaging (DWI); (**d**) high values on apparent diffusion coefficient (ADC) maps. In the dynamic gadolinium ethoxybenzyl diethylenetriamine pentaacetic acid-enhanced MRI (EOB-MRI), multiple masses were detected as hypo-intensity lesions both in the early phase (**e**) and hepatobiliary phase (**f**). Typical tumors are indicated with arrowheads.

Magnetic resonance imaging (MRI) showed hypo-intensity on T1-weighted imaging, high intensity on T2-weighted imaging, high intensity on diffusion-weighted imaging (DWI) and high values on apparent diffusion coefficient (ADC) maps. Dynamic gadolinium ethoxybenzyl diethylenetriamine pentaacetic acid-enhanced MRI (EOB-MRI) showed hypo-intensity in all (early, late and hepatobiliary) phases (Figure 4).

**Figure 5:**
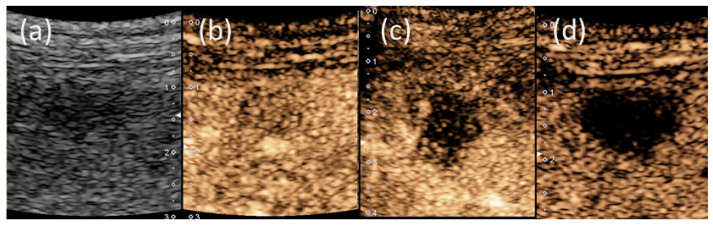
Findings of ultrasonography (US): (**a**) a hypoechoic tumor of 10 mm in size was observed (Aplio; Canon Medical Systems, Japan). On Sonazoid contrast-enhanced US, the tumor showed very slight hypoenhancement in the arterial phase (**b**), washout with peripheral enhancement in the portal phase (**c**) and hypointensity in the Kupffer phase (**d**).

**Figure 6:**
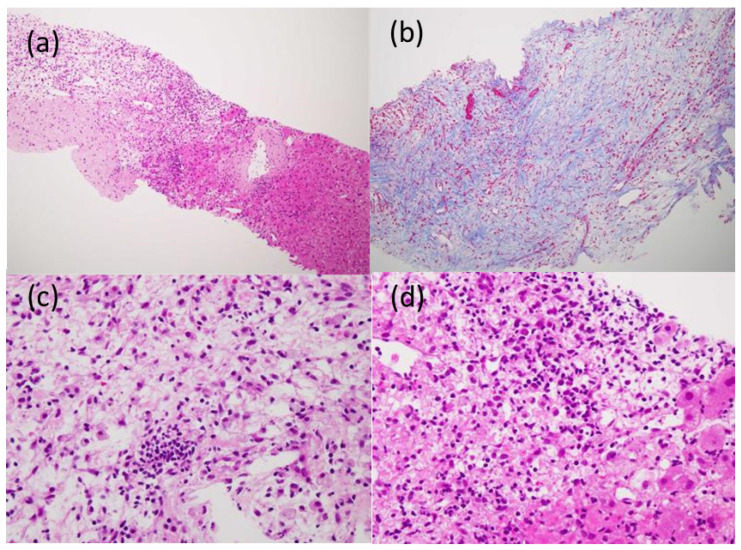
Histological findings obtained by the US-guided needle biopsy: shedding of liver cells, edematous changes and fibrosis with the infiltration of inflammatory cells, including lymphocytes and macrophages, were observed; (**a**) Hematoxylin and Eosin (HE) staining (×4); (**b**) Azan staining (×10); (**c**,**d**) HE staining (×20).

**Figure 7:**
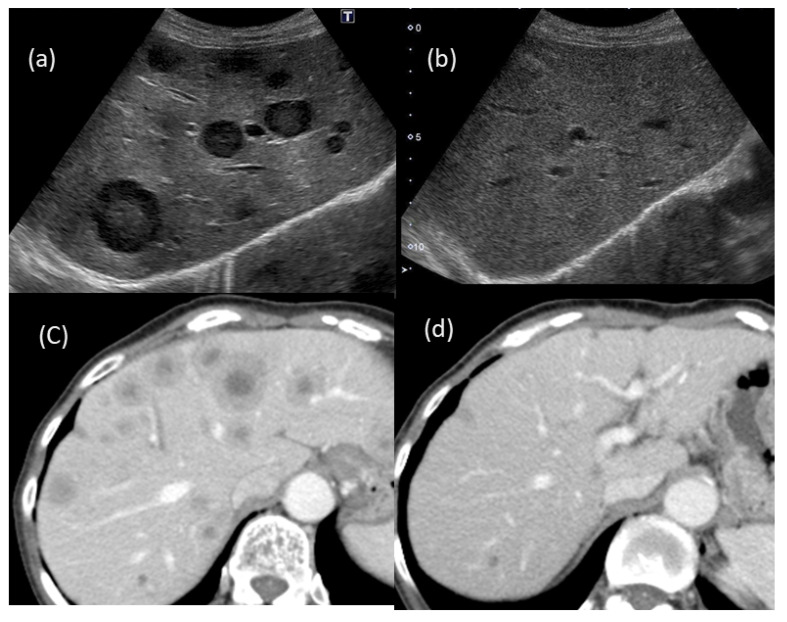
Changes in the US and CT findings: multiple tumors that had been suggested to be metastatic tumors (**a**,**b**) were barely detected after two months of observation; (**a**,**b**) US findings; (**c**,**d**) CT findings.

In the US findings (Aplio, Canon Medical Systems, Ohtawara, Tochigi, Japan), a hypoechoic tumor of 10 mm in size was observed (Figure 5a). On the Sonazoid contrast-enhanced US, the tumor showed very slight hypoenhancement in the arterial phase (Figure 5b), washout with peripheral enhancement in the portal phase (Figure 5c) and hypointensity in the Kupffer phase (Figure 5d). A percutaneous liver biopsy showed liver tissue with cell shedding, fibrotic changes, and infiltration of inflammatory cells, including lymphocytes and macrophages. There were no malignant findings. As a result, IPT was thus suggested (Figure 6). After two months of observation, the tumors spontaneously regressed and nearly vanished (Figure 7).

## 3. Discussion

IPT is a rare inflammation-related benign tumor with histopathological findings of the proliferation of myofibroblastic cells and the infiltration of inflammatory cells [11]. IPT has been most frequently reported as a lung tumor and rarely develops in the liver [1,2,3]. The etiology and pathogenesis of IPT are uncertain, although various factors, such as autoimmune disorders, infections, chronic biliary inflammation, and traumatic injury, have been suggested to be associated with its development [6,12,13,14]. However, few cases of IPT complicated with PBC or RA have been reported [9,11,15,16]. IPT is generally observed as a solitary lesion, and spontaneous regression is uncommon [4,5,6,7,8]. According to a paper by Yang et al. [7], 110 out of 114 cases had a solitary tumor, and only 4 cases had multiple tumors. Since IPT with multiple lesions is rare, we initially suspected that our patient suffered from metastatic tumors. Our report is unique in that multiple IPTs occurred in a PBC patient and spontaneously regressed.

IPTs show various symptoms and imaging findings [17,18,19]. In 2013, Watanabe et al. [17] reviewed 140 cases of IPT of the liver in Japan. As the chief complaints, 42.1% of cases cited abdominal pain and 15% cited general fatigue. In addition, 11.4% of the cases were detected incidentally at an annual examination. However, no commonly observed typical symptoms have been reported. In addition, IPT shows various imaging findings, depending on the proportions of fiber-like components and infiltrating inflammatory cells [12,18]. Regarding the CT findings, 86.2% of cases were detected as enhanced lesions [19]. Regarding the MRI findings [19], 86.4% of cases showed hypo-intensity lesions on T1-weighted imaging, and 76.2% showed high-intensity lesions on T2-weighted imaging. Recently, the utility of CEUS was reported [20]; however, decisive imaging findings of IPT have not been established. In addition, IPT can show an elevated standardized uptake value on FDG-PET [21]. Thus, it is difficult to distinguish IPTs from malignant tumors, and patients with hepatic IPTs often undergo surgical resection to obtain a final diagnosis [8,10,16,17,22,23]. 

In our case, CE-CT showed low-attenuation areas in the center of the tumors (Figure 2). However, at the early vascular phase of CEUS, the tumors were shown as very slight hypoenhancement with some hypervascular parts. These CEUS findings were inconsistent with those of metastatic liver tumors, which have internal necrosis. Malignant lesions reportedly show a high intensity on DWI and hypo-values on ADC because of their high cell density [24,25]. Regarding the MRI findings in our case, the DWI showed high-intensity lesions; however, high values were observed on the ADC maps. In addition, the FDG-PET showed a normal standardized uptake value for the tumors. These results were atypical for metastatic tumors. 

Despite the presence of findings suggestive of metastatic tumors (Figure 1 and Figure 2), we assessed several different types of clinical data available, such as different imaging techniques, and ultimately concluded that the tumors were benign. Thus, it is quite important to make a diagnosis by combining various imaging findings and considering the patient’s background and clinical course. 

In this case, we concluded that the tumors were benign, but we did not determine which type of cells proliferated and contributed to the uncommon findings of the patient. It would be interesting to investigate the characteristics of tumors with immunostaining of cell-specific markers, such as cytokeratin 8/18 (for hepatocytes) or cytokeratin 7/19 (for cholangiocytes). Moreover, it would be interesting to examine the phenotype of the infiltrating inflammatory cells by detecting specific markers for different cell types.

In summary, we presented a case of multiple IPTs of the liver that spontaneously regressed within two months. Physicians should avoid making a hasty judgment based solely on a few clinical findings.

## Figures and Tables

**Table 1:** Laboratory examinations: CRE: creatinine; ALP: alkaline phosphatase; γ-GTP: γ-glutamyl transpeptidase; TP: total protein; BS: blood sugar; CRP: C-reactive protein; ANA: antinuclear antibody; AMA-M2: anti-mitochondrial antibody-M2; CEA: carcinoembryonic antigen; CA19-9: carbohydrate antigen 19-9; AFP: α-fetoprotein; PIVKA-II: protein induced by vitamin K absence or antagonist-II. The lower and upper limits of the normal ranges are shown in parentheses (lower limit-upper limit).

WBC	6970	/μL	(4000–9000)	T-bil	0.3	mg/dL	(0.2–1.2)	PT-INR	1.06		(0.91–1.14)
Hb	11.7	g/dL	(11.5–15.0)	AST	37	U/L	(13–33)	PT%	90.5	%	(70–120)
Hct	36.3	%	(35.0–46.0)	ALT	20	U/L	(6–27)	IgM	112	mg/dL	(46–260)
Plt	279	×10^3^ /μL	(150–350)	LDH	387	U/L	(119–229)	IgG	931	mg/dL	(870–1700)
				ALP	374	U/L	(115–339)	ANA	×160		(<40)
Na	143	mmol/L	(138–146)	γ-GTP	30	U/L	(6–46)	AMA-M2	193	idx	(≤7.0)
K	3.4	mmol/L	(3.6–4.9)	Alb	3.1	g/dL	(3.7–4.7)	CEA	2.8	ng/mL	(≤5.0)
Cl	109	mmol/L	(99–109)	TP	6.0	g/dL	(6.6–8.7)	CA19–9	29.6	U/mL	(≤37.0)
BUN	7	mg/dL	(8–20)	BS	92	mg/dL	(70–109)	AFP	8.8	ng/mL	(≤10.0)
CRE	0.44	mg/dL	(0.36–1.06)	CRP	0.7	mg/dL	(≤0.3)	PIVKA-II	20	mAU/mL	(<40)

## Data Availability

Data supporting this case report are available from the corresponding author on reasonable request.
